# Predictive Factors of the Use of Rituximab and Belimumab in Spanish Lupus Patients

**DOI:** 10.3390/medicina59081362

**Published:** 2023-07-25

**Authors:** O. Capdevila, F. Mitjavila, G. Espinosa, L. Caminal-Montero, A. Marín-Ballvè, R. González León, A. Castro, J. Canora, B. Pinilla, E. Fonseca, G. Ruiz-Irastorza

**Affiliations:** 1 Autoimmune Diseases Unit, Department of Internal Medicine, Hospital Universitari de Bellvitge, L’Hospitalet de Llobregat, 08907 Barcelona, Spain; fmitjavila@bellvitgehospital.cat; 2 Bellvitge Biomedical Research Institute (IDIBELL), L’Hospitalet de Llobregat, 08907 Barcelona, Spain; 3 Department of Autoimmune Diseases, Hospital Clinic, 08036 Barcelona, Spain; gespino@clinic.cat; 4 Group of Basic and Translational Research in Inflammatory Diseases, Departament of Internal Medicine, Hospital Universitario Central de Asturias, Instituto de Investigación Sanitaria del Principado de Asturias (ISPA), 33011 Oviedo, Spain; lcaminal@yahoo.es; 5 Department of Internal Medicine, Hospital Clínico Universitario Lozano Blesa, 50009 Zaragoza, Spain; 6 Department of Internal Medicine, Hospital Universitario Virgen del Rocío, 41013 Seville, Spain; pacolageno@gmail.com; 7 Department of Internal Medicine, Hospital Universitari Sant Joan de Reus, 43204 Reus, Spain; 8 Department of Internal Medicine, Hospital Universitario de Fuenlabrada, 28942 Madrid, Spain; 9 Department of Internal Medicine, Hospital General Universitario Gregorio Marañón, 28007 Madrid, Spain; blancapinilla@telefonica.net; 10 Department of Internal Medicine, Hospital de Cabueñes, 33394 Gijón, Spain; evamfonseca@yahoo.es; 11 Autoimmune Diseases Research Unit, Department of Internal Medicine, BioCruces Bizkaia Health Research Institute, Hospital Universitario Cruces, UPV/EHU, 48903 Barakaldo, Spain

**Keywords:** systemic lupus erythematosus, belimumab, rituximab

## Abstract

*Objectives*: To analyze the characteristics and the predictive factors of the use of rituximab and belimumab in daily practice in patients from the inception cohort Registro Español de Lupus (RELES). *Material and methods*: The study included 518 patients. We considered patients treated with biologics who received at least one dose of rituximab or belimumab, and possible indications of those manifestations registered at the same time or in the previous 2 months of the start of the therapy. *Results*: In our cohort, 37 (7%) patients received at least one biological treatment. Rituximab was prescribed in 26 patients and belimumab in 11. Rituximab was mainly prescribed for hemolytic anemia or thrombocytopenia (11 patients, 42%), lupus nephritis and neuropsychiatric lupus (5 patients each, 19%). Belimumab was mostly used for arthritis (8 patients, 73%). In the univariate analysis, the predictive factors at diagnosis for the use of biologic therapy were younger age (*p* = 0.022), a higher SLEDAI (*p* = 0.001) and the presence of psychosis (*p* = 0.011), organic mental syndrome (SOCA) (*p* = 0.006), hemolytic anemia (*p* = 0.001), or thrombocytopenia (*p* = 0.01). In the multivariant model, only younger age, psychosis, and hemolytic anemia were independent predictors of the use of biologics. *Conclusions*: Rituximab is usually given to patients with hematological, neuropsychiatric and renal involvement and belimumab for arthritis. Psychosis, hemolytic anemia and age at the diagnosis of lupus were independent predictive factors of the use of biological agents. Their global effects are beneficial, with a significant reduction in SLE activity and a low rate of side effects.

## 1. Introduction

Systemic lupus erythematosus (SLE) is a heterogeneous autoimmune multisystemic disease, with a complex pathogenesis in which immune dysregulation plays an important role [1].

Signs and symptoms of SLE can affect a single organ or several organ systems, making it a difficult disease to diagnose. Typical manifestations include skin rashes, arthritis, serositis, and lupus nephritis. Hematological and neuropsychiatric involvement are less frequent. Early diagnosis of SLE is crucial to prevent flares and resultant tissue damage. Treatment response can be variable and difficult to predict. 

Despite the improvement in the prognosis of lupus within the last decades, the burden of disease is still determined by both the degree and the severity of the immunologic inflammatory disease and the resultant organ damage, either caused by the disease itself, by comorbidities, and/or by treatments [2,3]. Sustained remission is an important goal.

With the aim of achieving better control of disease activity, new therapeutic alternatives to glucocorticoids and immunosuppressants are being developed. A better understanding of the etiopathogenesis of SLE has led to the introduction of a number of biologic agents that specifically target disease pathways underlying the development and progression of lupus [4,5]. Some of these therapies, such as rituximab and belimumab, are available in clinical practice, while others are being tested in ongoing clinical trials. The use of such biologic agents is recommended in patients with an inadequate response to standard therapies [6]. 

Rituximab (RTX) is a chimeric mAb that targets CD20, a transmembrane protein on all B cells except pro-B cells and plasma cells, which results in cytotoxicity and B cell depletion [7]. Several case series and retrospective studies have shown improvement in SLE parameters, including lupus nephritis, despite negative results of randomized controlled trials (RCT) [8,9]. RTX efficacy was studied in nonrenal SLE with moderate to severe disease activity and is used off-label in refractory and relapsing SLE based on several observational nonrandomized studies [10,11].

Belimumab is a recombinant, fully human monoclonal antibody (mAb) that blocks the binding of soluble B lymphocyte stimulator to its receptor on B cells, thus, decreasing B cell survival, differentiation, and activation. It was the first biologic to be FDA-approved for SLE and is available as an i.v. infusion or a subcutaneous injection. In several large double-blinded phase III randomized controlled trials RCT, it has been shown to improve musculoskeletal and mucocutaneous manifestations and immunologic parameters in patients with active disease on background standard-of-care therapy. These studies initially excluded severe renal and central nervous system (CNS) forms [12,13,14]. A recent trial has also demonstrated its beneficial effect on lupus nephritis when added to standard treatment [15]. More recently belimumab has shown efficacy in decreasing SLE exacerbations and reducing glucocorticoid doses, thus, contributing to decreased damage accrual [16,17].

The aim of the present study is to analyze the use of these biologic agents in daily practice in our setting. RELES (Registro Español de Lupus Eritematoso Sistémico) is the first Spanish multicentric inception lupus cohort, a research project of the Group of Autoimmune Diseases within the Spanish Society of Internal Medicine, in which patients with a new diagnosis of SLE have been included since January 2009. Thus, we analyze the indications, baseline predictive factors, efficacy, and side effects of the use of biologic therapy in the RELES cohort. 

## 2. Materials and Methods

A total of 518 patients were enrolled in RELES by the end of 2020. Among them, 425 had completed at least one year of follow up, 371 two years, 268 three years, and 200 four years or more. All patients were attended at Internal Medicine Services of 44 Spanish public hospitals. Patients were enrolled at the time when at least 4 ACR classification criteria were met [18].

Recruitment started in January 2009 and data were prospectively collected and entered in a computerized database. All patients signed an informed consent document at the time of enrolment. The study protocol has been approved by the institutional research ethics boards of the coordinating center (Hospital Universitario Cruces) and all participating centers.

Information on demographic characteristics, clinical manifestations, laboratory results, disease activity measured by the Safety of Estrogens in Lupus Erythematosus National Assessment (SELENA) version of the SLE Disease Activity Index (SLEDAI) [19], and treatments received are registered at the time of enrolment and yearly thereafter. Damage accrual, measured by the Systemic Lupus International Collaborating Clinics (SLICC)/American College of Rheumatology (ACR) Damage Index (SDI) [20], is first recorded after 6 months of enrolment and yearly thereafter. 

For the purposes of this study, we considered patients treated with biologics who received at least one dose of rituximab or belimumab. We considered as possible indications of a biological treatment those manifestations registered at the same time or in the previous 2 months of the start of the therapy. We considered biologics-related infections to be those diagnosed within the first year after the administration of rituximab or belimumab. 

### Statistical Analysis

Descriptive data were generated using percentages, means, and standard deviations (SD). Such data included baseline demographic characteristics, clinical manifestations, and immunological profiles at baseline. Likewise, the indications for biologic use, as defined in the previous section, were summarized. 

The comparison of data from patients receiving or not receiving biologic therapy was performed using the Chi-square test, Fisher’s exact test, non-paired Student’s t-test, or Mann–Whitney test, as appropriate. Those variables with a *p* value of 0.2 or less in the univariate analysis were subsequently included in a logistic regression model with a backward stepwise selection of variables, in order to identify independent predictive factors at baseline for the use of biologic drugs during the follow up. 

The efficacy of biologic therapy was assessed by comparing mean SLEDAI scores just before and 6 months after starting therapy in patients receiving these drugs by paired T-test. Finally, infections after biologic therapy were summarized, and the proportions of treated and untreated patients suffering infections within the same period were compared by a Chi-square test.

All statistical analyses were performed using the IBM SPSS statistics19 software package for Windows.

## 3. Results

### 3.1. Baseline Clinical Characteristics at Diagnosis

A total of 518 patients were included in this study. The main clinical characteristics of the cohort and treatments received are shown on Table 1**.** Overall, 89% of patients were women and 78% were Caucasians. The mean SLEDAI score at diagnosis was 9. Most patients had mucocutaneous (75%) or articular manifestations (78%) followed by hematological disorders (21%), nephritis (19%), and serositis (17%).

### 3.2. Main Indications of Biologic Therapies and Concomitant Treatments

In our prospective cohort, 37 (7%) patients received at least one biological treatment. Rituximab was prescribed in 26 patients and belimumab in 11. Six patients received rituximab and belimumab consecutively during the study period. In three patients, treatment was administered for refractory diseases, and the other three received rituximab and belimumab for different organ manifestations.

Rituximab was mainly prescribed for hemolytic anemia and/or thrombocytopenia (11 patients, 42%), followed by lupus nephritis and neuropsychiatric lupus (5 patients each, 19%). Belimumab was mostly used for arthritis in the vast majority of patients (eight patients, 73%). The detailed indications for rituximab and belimumab are shown in Table 2.

The mean (SD) time from disease onset to the administration of the biologic treatment was 28 (30) months, 25 (27) months for rituximab, and 38 (33) months for belimumab. Rituximab was administered after a mean of 18, 23, and 39 months after the diagnosis of SLE, respectively, for hematological, neuropsychiatric, and renal involvement, whereas belimumab was administered after a mean of 30 months after the SLE diagnosis for articular symptoms.

All patients received glucocorticoids simultaneously to the biologic treatment, while 92% received hydroxychloroquine and 84% received immunosuppressants (5 azathioprine, 18 mycophenolate, 5 methotrexate, and 3 cyclophosphamide).

### 3.3. Predictive Factors at Baseline for the Use of Biologic Therapy

In the univariate analysis, the predictive factors at baseline for the eventual use of biologic therapy were younger age (33 vs. 40 years in patients not given biologics, *p* = 0.006), a higher SLEDAI score (14 vs. 9, respectively, *p* = 0.001), and the presence of psychosis, organic mental syndrome (SOCA), hemolytic anemia, or thrombocytopenia (Table 3). In the multivariate model, younger age, psychosis, and hemolytic anemia were independent predictors of the use of biologics (Table 4). 

### 3.4. Efficacy and Safety of Biologic Treatment

Regarding efficacy, we observed a significant reduction in lupus activity according to the mean (SD) SLEDAI scores before and after the administration of biologic treatment, namely 12.9 (8.6) vs. 4.4 (4.7), respectively (*p* < 0.001). 

Eleven (28%) patients suffered infections after the administration of biologic treatment: three patients had herpes zoster, seven had bacterial infections (four urinary tract infections, two had pneumonia, and one had pelvic inflammatory disease), while one had a cutaneous leishmaniasis. None of these conditions were lethal. The proportion of patients with infections within the same time span was similar in patients who did not receive biologic treatment (128/481 patients, 27%, *p* = 0.85). 

## 4. Discussion

In our prospective cohort, 7% of patients received biologic agents within the first 5 years of the disease course. Data on the number of patients with SLE requiring treatment with biologic agents are scarce. A French registry showed that 136/2551 patients (5.4%) received at least one dose of rituximab [21]. Even taking into account that our cohort includes patients who received either rituximab or belimumab, our results do not greatly differ from those reported. 

Younger age, psychosis, and hemolytic anemia at the time of diagnosis of SLE were the only independent predictive factors of the use of biologics in our cohort. In fact, hematological and neuropsychiatric lupus were also two of the main indications for rituximab, which, although not considered a first-line treatment, is being increasingly used in these scenarios due to the frequent lack of effectiveness of usual therapies [6].

It is noteworthy that although belimumab is the only biologic treatment approved for lupus, rituximab was used more frequently and earlier than belimumab in our cohort. Rituximab is usually prescribed for refractory or relapsing severe lupus manifestations, which also include nephritis and arthritis apart from the already mentioned neuropsychiatric and hematological manifestations [22,23]. It is important to remark that rituximab was given earlier within the course of disease to patients with hematological manifestations than to those with neuropsychiatric lupus or nephritis. This is probably due to the fact that usual first-line therapies are less effective in immune thrombocytopenia and hemolytic anemia [22,24]. The role of rituximab in neuropsychiatric lupus is not well defined [25,26], while it is clearly considered a rescue therapy in lupus nephritis [27,28]. 

In our cohort, belimumab was mainly used for arthritis. Standard treatments for patients with arthritis and mucocutaneous manifestations include hydroxychloroquine and low-dose prednisone, with immunosuppressants being added in refractory cases or when glucocorticoid maintenance doses cannot be reduced [29]. Recent data could suggest that an earlier use of belimumab in the SLE course could speed up the clinical response, especially in patients with a relapsing–remitting pattern who are taking high prednisone doses [30,31]. 

It well stablished that modifying the disease course with effective therapies and steroid-sparing regimens may reduce organ damage, improve outcomes and decrease mortality in patients with SLE [16]. However, what should be the role of biologic therapy within the global therapeutic strategy of lupus is not well defined. High early and sustained response rates with “conventional” therapy based on pulses of methyl-prednisolone and reduced doses of oral prednisone have been recently shown [32]. In the RELES cohort, patients receiving biologic therapy had a significantly more active disease at diagnosis and a high concomitant use of other therapies, all suggesting refractory disease. After starting biologics, a significant decrease in activity, as measured by SLEDAI scores, was accomplished. These results support the current EULAR recommendations for the use of belimumab and rituximab, both as a second-line therapy in patients who are refractory or intolerant to non-biologic therapy [6]. It must be remarked upon once more that hydroxychloroquine has convincingly shown long-term effects in reducing damage accrual and improving survival, so its role as a universal therapy for lupus patients cannot be replaced at this time by any other therapy, including biologics [33], despite the promising long-term effects of belimumab [32]. Our data are also reassuring in showing that biologic agents were not associated with an increase in the number of infections, as previously described by other authors [9,21].

We acknowledge some limitations of this study. This study is based on a multicenter Spanish register and, therefore, it does not include a control group comparing the use of biological treatment with the standard of care. The use of biologics was decided by the physicians caring for the patients, without any pre-specified protocol. The main outcome measure was the reduction in the SLEDAI score, without specific data on the evolution of the clinical manifestation leading to the use of biologics. The low number of biologic-treated patients and the diversity of indications made it impossible to offer a detailed statistical analysis on this issue. The long-term effects on damage and glucocorticoid use have not been addressed due to the low numbers of biologic-treated patients with prolonged follow-up. 

On the other hand, our real-world data offer a realistic view of the use of rituximab and belimumab in our setting. The high proportion of those biologic-treated patients being on prednisone, hydroxychloroquine, and immunosuppressive drugs point to a second-line indication for patients who are refractory to conventional therapy, thus, following current guidelines [6]. Our study has revealed the main baseline predictors for the use of either rituximab or belimumab, as well as global beneficial effects on lupus activity in patients who are refractory to other therapies. 

## 5. Conclusions

In summary, our study reveals that younger age, neuropsychiatric lupus, and hemolytic anemia at SLE diagnosis predict the use of rituximab or belimumab at some point of their SLE course. The global effects of both drugs are beneficial in these groups of patients, with a significant reduction in SLE activity and a low rate of side effects. 

## Figures and Tables

**Table 1:** Baseline clinical characteristics of the Registro Español de Lupus Eritematoso Sistémico (RELES) cohort.

	No Biologics (*n* = 479)	Rituximab (*n* = 26)	Belimumab (*n* = 11)
**Age at disease onset, mean (SD)**	40 (16)	33 (14)	32 (16)
**Female, *n* (%)**	426 (89)	23 (88)	11
**Caucasian, *n* (%)**	372 (78)	19 (73)	10 (91)
**Hispanic, *n* (%)**	95 (20)	5 (19)	1 (9)
**Asian, *n* (%)**	7(1)	2(8)	
**Afro-American (%)**	5 (1)		
**Cutaneous disease, *n* (%)**	360 (75)	19 (73)	10 (91)
**Arthritis, *n* (%)**	370 (77)	20 (77)	11
**Neurologic disease, *n* (%)**	29 (6)	6 (23)	1 (9)
Seizures	7 (1)	0	1 (9)
Psychosis	4 (1)	3 (11)	0
Organic mental syndrome	0	2 (8)	0
Myelitis	4 (1)	1 (4)	0
**Serositis, *n* (%)**	83 (17)	4 (15)	1 (9)
Pleuritis	67 (14)	3 (11)	0
Pericarditis	50 (10)	4 (15)	1 (9)
**Pneumonitis, *n* (%)**	15 (3)	2 (8)	0
**Glomerulonephritis, *n* (%)**	86 (18)	9 (35)	2 (18)
Proliferative glomerulonephritis	51 (11)	5 (19)	1 (9)
**Hematological, *n* (%)**	86 (18)	15 (58)	5 (45)
Hemolytic anemia	33 (7)	10 (38)	4 (36)
Thrombocytopenia	65 (14)	11 (42)	1 (9)
**SLEDAI, mean (SD)**	9 (7)	15 (11)	12 (7)

**Table 2:** Indications for rituximab and belimumab.

SLE Manifestations	Rituximab (*n* = 26)	Belimumab (*n* = 11)
Arthritis	2(8%)	8 (73%)
Hematological	11 (42%)	0
Neuropsychiatric disease	5 (19%)	1 (9%)
Serositis	0	1 (9%)
Proliferative glomerulonephritis	5 (19%)	1(9%)
Pneumonitis	3 (11%)	0

**Table 3:** Predictive factors at baseline associated with the use of biologic treatment.

	Biologic Treatment (*n* = 37)	No Biologic Treatment (*n* = 481)	*p*
**Age at disease onset, mean (SD)**	33 (14)	40 (17)	**0.006**
**Female, *n* (%)**	34 (92)	427 (89)	0.79
**Caucasian, *n* (%)**	29 (78)	374 (78)	1
**SLEDAI, mean (SD)**	14 (10)	9 (7)	**0.001**
**Cutaneous disease, *n* (%)**	28 (76)	362 (75)	1
**Arthritis, *n* (%)**	31 (84)	372 (77)	0.42
**Neurological disease, *n* (%)**	7 (19)	29 (6)	**0.01**
Seizures	1 (3)	7 (2)	0.45
Psychosis	3 (8)	4 (1)	**0.01**
Organic mental syndrome	2 (5)	4 (1)	**0.06**
Myelitis	1 (3)	4(1)	0.31
**Serositis, *n* (%)**	5 (14)	84 (17)	0.65
Pleuritis	3 (8)	68 (14)	0.45
Pericarditis	5 (13)	51 (11)	0.58
**Pneumonitis, *n* (%)**	2 (5)	15 (3)	0.34
**Nephritis, *n* (%)**	11(30)	86 (18)	0.08
Proliferative nephritis	6 (16)	51 (11)	0.27
**Hematological, *n* (%)**	20 (54)	87 (18)	**<0.001**
Hemolytic anemia	14 (38)	34 (7)	**<0.001**
Thrombocytopenia	12 (32)	65 (14)	**0.006**

**Table 4:** Multivariant analysis of predictive factors for the use of biologics at diagnoses.

	Initial Model OR (95% CI)	Final Model OR (95% CI)
**Age at disease onsett**	1.031 (1.007–1.056)	0.970 (0.945–0.995)
**Organic mental syndrome**	0.147 (0.026–0.828)	
**Psychosis**	0.095 (0.020–0.442)	11.07 (1.885–65.07)
**Hemolytic anemia**	0.125 (0.059–0.265)	7.283 (3.164–16.767)
**Thrombocytopenia**	0.326 (0.156–0.680)	
**Lupus nephritis**	1.938 (0.922–4.073)	
**SLEDAI**	0.939 (0.908–0.971)	

## Data Availability

The data used during the current study is available from the corresponding author on reasonable request.
